# How hydrolytic exoribonucleases impact human disease: Two sides of the same story

**DOI:** 10.1002/2211-5463.13392

**Published:** 2022-03-20

**Authors:** Susana M. Costa, Margarida Saramago, Rute G. Matos, Cecília M. Arraiano, Sandra C. Viegas

**Affiliations:** ^1^ Instituto de Tecnologia Química e Biológica António Xavier Universidade Nova de Lisboa Oeiras Portugal

**Keywords:** Dis3L2, human disease, pathogens, RNA decay, RNase II/RNB family, viral exoribonuclease

## Abstract

RNAs are extremely important molecules inside the cell, which perform many different functions. For example, messenger RNAs, transfer RNAs and ribosomal RNAs are involved in protein synthesis, whereas noncoding RNAs have numerous regulatory roles. Ribonucleases (RNases) are the enzymes responsible for the processing and degradation of all types of RNAs, having multiple roles in every aspect of RNA metabolism. However, the involvement of RNases in disease is still not well understood. This review focuses on the involvement of the RNase II/RNB family of 3′–5′ exoribonucleases in human disease. This can be attributed to direct effects, whereby mutations in the eukaryotic enzymes of this family [defective in sister chromatid joining (Dis3; or Rrp44), Dis3‐like exonuclease 1 (Dis3L1; or Dis3L) and Dis3‐like exonuclease 2 (Dis3L2)] are associated with a disease, or indirect effects, whereby mutations in the prokaryotic counterparts of RNase II/RNB family (RNase II and/or RNase R) affect the physiology and virulence of several human pathogens. In this review, we compare the structural and biochemical characteristics of the members of the RNase II/RNB family of enzymes. The outcomes of mutations impacting enzymatic function are revisited, in terms of both the direct and indirect effects on disease. Furthermore, we also describe the severe acute respiratory syndrome coronavirus 2 (SARS‐CoV‐2) viral exoribonuclease and its importance to combat the COVID‐19 pandemic. As a result, RNases may be a good therapeutic target to reduce bacterial and viral pathogenicity. These are the two perspectives on RNase II/RNB family enzymes that are presented in this review.

## Graphical abstract

Ribonucleases (or simply RNases) are the enzymes responsible for the processing and degradation of all types of RNAs, having multiple roles in every aspect of RNA metabolism. Here, we review what is known about the involvement of the RNase II/RNB family of 3′–5′ exoribonucleases in human disease. As RNases may constitute an efficient approach to fight pathogenicity, this is also discussed in the context of bacterial and viral infections.

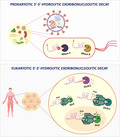

**Abbreviations**
CSD: cold‐shock domain
Dis3: defective in sister chromatid joining
Dis3L1: Dis3‐like exonuclease 1
Dis3L2: Dis3‐like exonuclease 2
MLS: Marfan‐like syndrome
nsp10: nonstructural protein 10
nsp14: nonstructural protein 14
PIN: PilT N‐terminus domain
PRLMNS: Perlman syndrome
RNase: ribonuclease
RNB: ribonuclease B (renamed as RNase II) catalytic domain
SARS‐CoV‐2: severe acute respiratory syndrome coronavirus 2
WT: Wilms' tumour

The amount of RNA present in the cell is modulated by cellular needs and environmental demands. The final RNA levels are determined by the balance between RNA synthesis and degradation. Ribonucleases (RNases) are the enzymes responsible for the degradation of all types of RNA, ensuring RNA processing and turnover [1, 2]. RNases also allow the recycling of ribonucleotides and participate in several quality control mechanisms, eliminating aberrant RNAs to safeguard the fidelity of gene expression. Therefore, these enzymes serve multiple roles in every aspect of RNA metabolism. There are two types of RNases in the cell: the endoribonucleases, which cleave the RNA molecule internally, and the exoribonucleases, which degrade the RNA starting from one of its extremities.

Much research has been focused on the characterization of RNases. Many are known (e.g. about 20 RNases in *Escherichia coli*), but only a few have an essential activity [3, 4]. RNases are generally redundant, acting in a concerted overlap that allows fine‐tuning of their activities. They can act alone, or they can cooperate in RNA degradation complexes (such as the prokaryotic degradosome [5, 6, 7, 8] and the eukaryotic exosome [9, 10, 11]) in which exo‐ and endonucleolytic activities are coordinated.

Focusing on the exoribonucleolytic activity, bacteria have two main distinct 3′–5′ exoribonuclease activities: one is catalysed by RNase II or RNase R, which are processive hydrolytic exonucleases of the RNase II/RNB family of enzymes; and the second is performed by the phosphorolytic polynucleotide phosphorylase (PNPase). The key features of RNA degradation have been conserved throughout evolution, and the mechanisms of RNA degradation in bacteria and eukaryotes rely on the same basic set of enzymatic activities. As an example, the eukaryotic exosome has a PNPase‐like core structure, although it has evolved in terms of complexity [9, 12, 13]. In some eukaryotes, the exosome lost its phosphorolytic activity, and the only known catalytically active RNase components are the hydrolytic exoribonucleases from the RNase D (Rrp6; EXOSC10 in humans) and/or RNase II/RNB families of enzymes, with the ones from the last family being the major contributors to this activity. Eukaryotes may express three different homologues of the RNase II/RNB family: defective in sister chromatid joining (Dis3; or Rrp44), Dis3‐like exonuclease 1 (Dis3L1; or Dis3L) and Dis3‐like exonuclease 2 (Dis3L2). In the last decade, these eukaryotic members of the RNase II/RNB family of enzymes (Dis3‐like enzymes) have been in the spotlight due to their implication in important human diseases (reviewed in [14, 15]): for instance, multiple myeloma (associated with specific mutations in hDIS3 and the intracellular ratio of its two isoforms) [16] or cerebellar medulloblastoma (related to hDIS3L1 expression levels) [17]. In turn, the involvement of hDIS3L2 in several human diseases, including Perlman syndrome (PRLMNS), will be addressed in further detail in this review.

Regarding the prokaryotic counterparts of the family, different publications have demonstrated the role of these exoribonucleases in the establishment of virulence in Gram‐negative pathogens (reviewed in [18]). For instance, RNase R has been associated with virulence, since it impacts growth, motility, competence and adaptation to cold, among other physiological processes (see below).

In this article, we review some of the structural and biochemical characteristics of members of this family of enzymes, and the physiological implications of alterations in their activity linked with disease. We present how mutations in these enzymes are directly related to human disease or may impact on the virulence of human pathogens. We will describe the prokaryotic RNases, as they include the prototype member of the RNase II/RNB family (RNase II), and we also focus on the complexity of the eukaryotic family members. Lastly, a brief contextualization of a viral hydrolytic exoribonuclease is included to emphasize the extreme importance of studying 3′–5′ exoribonucleolytic RNA degradation in the context of disease and infection.

## Prokaryotic RNA decay

**Fig. 1:**
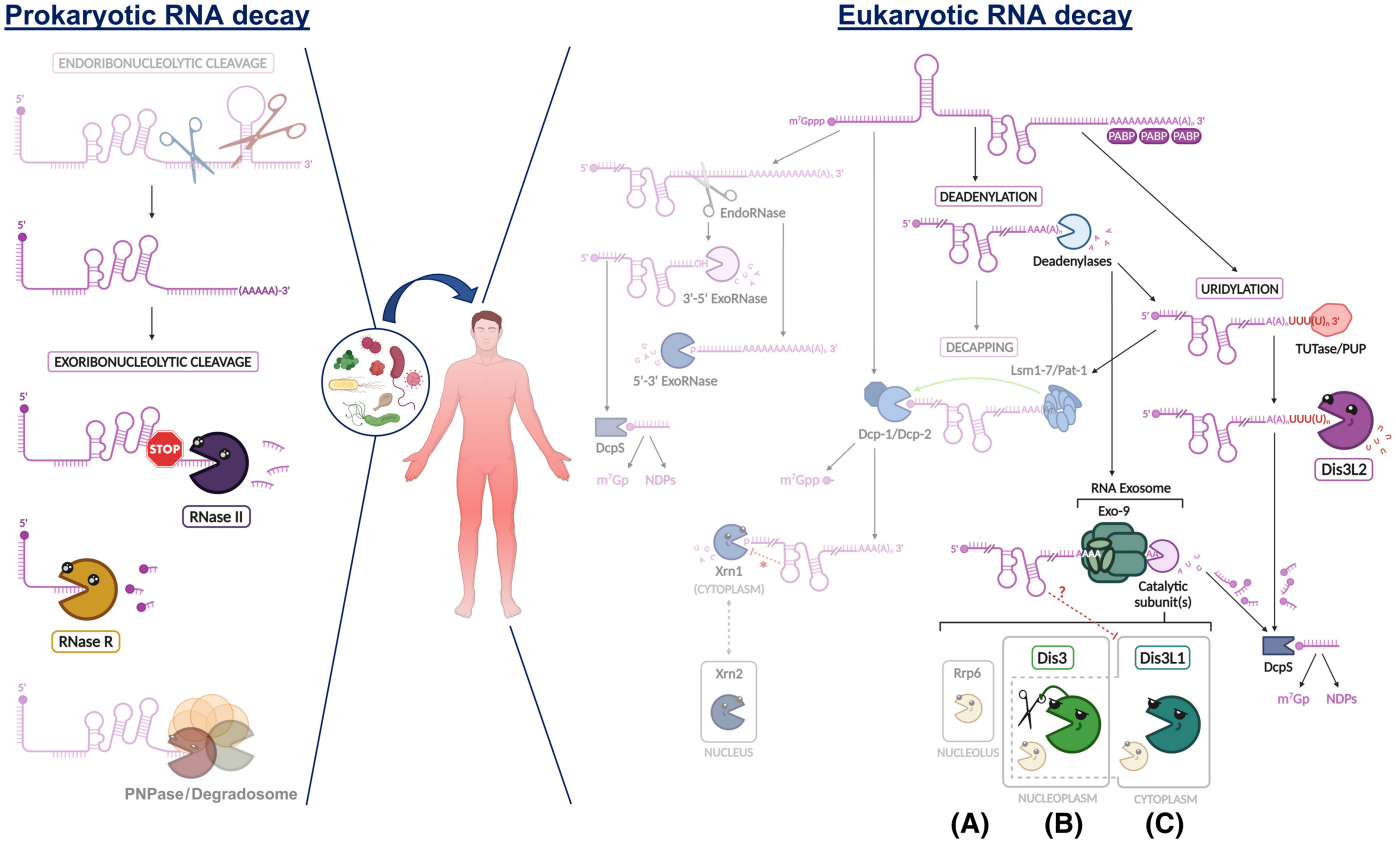
3′–5′ RNA decay (via hydrolysis) in prokaryotic and eukaryotic human cells. Left panel: In bacteria, the decay of the majority of RNA transcripts starts with an endoribonucleolytic cleavage (e.g. RNase E; faded image). After this initial step, the breakdown products can be hydrolytically degraded by 3′–5′ exoribonucleases from the RNB family of enzymes (RNase II and/or RNase R). This exoribonucleolytic activity can be promoted by the 3′‐polyadenylation of RNAs. RNase II accounts for 90% of RNA degradation in Gram‐negative bacteria, and it is sensitive to secondary structures. RNase R is a cold‐shock protein able to degrade structured RNA transcripts. Both enzymes generate end products of 4 and 2 nts, respectively. 3′–5′ exoribonucleolytic RNA degradation can be phosphorolytic (faded image). In that case, PNPase acts alone or together with other enzymes in a degradation complex called the degradosome. Right panel: In eukaryotic cells, mature cytoplasmic mRNAs are usually stabilised by a 5′ cap and a long 3′ poly(A) tail, to which poly(A) binding proteins (PABPs) may bind, thereby protecting that extremity from degradation. If mRNAs possess premature termination codons, they will probably be cleaved by an endoribonuclease (endoRNase, represented in the pathway on the left of the figure), with the resulting fragments being further digested by exoribonucleases (exoRNases) starting on the extremities generated by the internal disruption of the transcript. However, mRNA decay starts more frequently through partial or complete deadenylation (at the top centre of the figure), which is carried out by deadenylases (e.g. Ccr4‐Not and Pan‐2/Pan‐3 complexes). Subsequently, deadenylated mRNAs can be further decapped (represented at the bottom centre of the figure) or be readily degraded in the 3′–5′ direction by the RNA exosome. Decapping is stimulated by the Lsm1‐7/Pat‐1 complex, which recruits the decapping complex Dcp‐1/Dcp‐2. This step enables degradation in the 5′–3′ direction by enzymes of the 5PX superfamily: Xrn1 in the cytoplasm or Xrn2 in the nucleus. ‘*’ indicates that, despite Xrn1 being able to unwind highly structured RNA molecules, some of them block Xrn1 progression. The RNA exosome has distinct isoforms, which exhibit differences in cellular localization: (a) in the nucleolus, the Exo‐9 inert core binds to Rrp6 (an exoRNase from *Escherichia coli* RNase D family); (b) in the nucleoplasm, the major catalytic subunit is Dis3 (an exoRNase, which also possesses endoRNase activity and belongs to the RNB/RNase II family); (c) in the cytoplasm, the major subunit is Dis3L1 (an exoRNase of the RNB/RNase II family), but the nucleoplasmic isoform of the RNA exosome (with Dis3 instead of Dis3L1 enzyme associated with Exo‐9) may also be present in smaller amounts (dashed grey line in the box). Of note, Rrp6 is known to be present in all human isoforms of the RNA exosome, but it is absent in the cytoplasmic isoform of yeast. ‘?’ means there is still no evidence that Dis3L1 degrades secondary structures. Apart from the abovementioned pathways, transcripts can be targeted for uridylation by terminal uridylyltransferases (TUTases) or poly(U) polymerases (PUPs; on the right). This 3′‐end modification specifically recruits the Dis3L2 enzyme (an exoRNase from the RNB/RNase II family) that further degrades the molecule in the 3′–5′ direction. Small, capped degradation products are fully hydrolysed by DcpS. The faded parts of the image represent mechanisms of RNA decay not explored in this review. Created with BioRender.com.

In the model bacterium *E. coli*, RNA decay usually begins with an endonucleolytic cleavage at one or more internal sites on the RNA molecule (Fig. 1) [19, 20]. After endonucleolytic cleavage, breakdown products are ready for exonucleolytic digestion. Three exoribonucleases are mainly involved in RNA decay in *E. coli*: PNPase, RNase R and RNase II (Fig. 1). All of these enzymes degrade RNA processively and nonspecifically from the 3′‐end. While PNPase is a phosphorolytic exoribonuclease yielding nucleoside diphosphates as reaction products, both RNase R and RNase II catalyse the hydrolysis of RNA substrates, producing nucleoside monophosphates [2, 21, 22, 23, 24].

Unlike RNase R, which is able to digest structured RNA [25], the degrading activity of RNase II and PNPase is sensitive to secondary structures. However, PNPase can also proceed through extensive folded RNA when acting in association with other proteins. Its association with the helicase RhlB or integration into the degradosome allows the unwinding of the RNA stem–loops [6, 26, 27].

## Eukaryotic RNA decay

In eukaryotes, mature mRNAs usually present a 5′ cap and a 3′‐terminal poly(A) tail. These molecules can be destroyed through several pathways, involving either endo‐ or exoribonucleases (Fig. 1) [28]. The first step in these pathways is commonly the deadenylation or decapping of the transcripts. The deadenylation consists of the loss or shortening of the poly(A) tail [29]. In turn, decapping is triggered by the binding of the Lsm1–7/Pat‐1 complex (decapping activator, consisting of the Pat‐1 protein that recruits Sm‐like proteins Lsm1 to Lsm7) preferably to the 3′ extremity of deadenylated mRNAs, blocking the access of exoribonucleases to this RNA terminal [30]. Next, the Dcp‐1/Dcp‐2 complex (composed of decapping enzymes 1 and 2) promotes 5′ cap removal, leading to the exposure of the 5′‐end of the transcript [31, 32].

The degradation can then proceed in two pathways. If decapping occurs, then decay proceeds in the 5′–3′ direction: the 5′–3′ exoribonuclease 1 (Xrn1, also known as Pacman) is able to completely degrade the RNA starting from its 5′‐monophosphate end [33, 34]. While Xrn1 is cytoplasmic, this same function is performed by Xrn2 (also known as Rat1) in the nucleus [28, 35, 36]. On the contrary, if the mRNA is just deadenylated, then the RNA exosome is capable of attaching to the mRNA 3′‐end to degrade the molecule in the 3′–5′ direction [29, 37]. Depending on the cellular location, the nine‐subunit exosome core is associated with Rrp6 (known as PM/Scl‐100 or EXOSC10 in humans), Dis3 (also known as Rrp44 or Tazman) or Dis3L1 exoribonucleases. Rrp6 belongs to the RNase D family, and it is the only catalytic subunit of the RNA exosome in the nucleolus. Both Dis3 and Dis3L1 proteins are from the RNase II/RNB family of exoribonucleases and constitute the most prominent catalytic subunits of the RNA exosome in the nucleoplasm and cytoplasm, respectively. In any case, after digestion by the RNA exosome, the 5′ cap in the remaining fragment (usually smaller than 10 nucleotides) is eventually decomposed by the decapping scavenger (DcpS) enzyme [38, 39].

The above are the most widely studied general processes of RNA decay. However, the discovery of Dis3L2, another member of the RNase II/RNB family, revealed a new alternative eukaryotic RNA decay pathway independent of deadenylation and decapping, but rather mediated by uridylation. This major finding took place in fission yeast [40], and almost simultaneously in mice [41] and humans [42, 43], and challenged all the previous assumptions of eukaryotic RNA degradation pathways. It is worth highlighting some key cellular processes for which the Dis3L2‐dependent degradation pathway is essential. These include the role of Dis3L2 in ribosomal RNA (rRNA) biogenesis [44], endoplasmic reticulum‐associated mRNA translation [45], cell differentiation [41, 43, 46] and proliferation [47, 48], T‐cell activation [49] and spermatogenesis [50]. It may also be implicated in mitosis [51, 52] and apoptosis [53, 54, 55]. Furthermore, Dis3L2 is involved in the quality control mechanism of nonsense‐mediated decay (NMD), which is triggered by the presence of premature translation–termination codons (PTCs) in mRNAs [56, 57].

## The RNase II/RNB family of enzymes: a widespread family of 3′–5′ exoribonucleases

The RNase II/RNB family of enzymes display hydrolytic 3′–5′ exonucleolytic activity, and they are present in all domains of life. Changes in their catalytic functions or alterations in their cellular levels can relate to pathogenesis and disease. As such, we will revisit the main catalytic properties of these enzymes to contextualize their importance.

In prokaryotes, the representative enzymes of this family are RNase II and RNase R. Both are key players in RNA degradation and quality control, being present in a wide range of bacteria, including pathogenic agents. In what concerns their catalytic properties [58], both proteins differ in the size of the final degradation products released, and in their ability to unwind and digest structured RNAs. RNase II releases a 4‐nucleotide (nt) end product [59, 60], stalling 5–7 nucleotides before RNA double‐stranded regions [60], while RNase R generates 2‐nt end products and is able to cleave double‐stranded molecules if they have at least a 5‐nt single‐stranded overhang in their 3′‐end [25]. In turn, eukaryotes may express three different homologues of RNase II/RNB family: Dis3 (or Rrp44), Dis3L1 (or Dis3L) and Dis3L2 (see below).

**Fig. 2:**
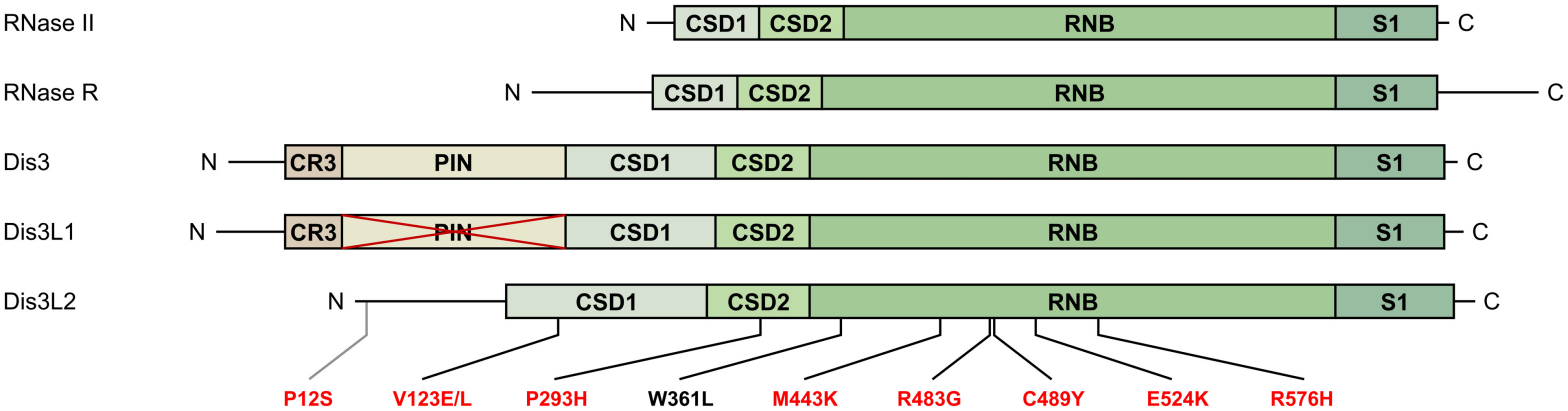
Protein domain organization of enzymes from the RNase II/RNB family. Representation of protein domains present in representative members of the RNase II/RNB family: bacterial RNase II and RNase R, and eukaryotic Dis3 (or Rrp44), Dis3L1 (or Dis3L) and Dis3L2. While the PIN domain confers an additional endonucleolytic activity to Dis3, it is inactive in Dis3L1 (depicted by a cross on the domain). The position of the amino acid substitutions in the hDIS3L2 protein that have been linked with human diseases (as detailed in Table 1) is indicated. The amino acid substitutions associated with PRLMNS/WT are denoted in red; the amino acid substitution associated with ATC is denoted in black. C, carboxyl terminus; N, amino terminus. The dimensions of the domains are approximately to scale.

Notably, all members of the RNase II/RNB family have a comparable protein domain organization (Fig. 2). From the amino terminus (N‐term) to the carboxyl terminus (C‐term), the domains that usually compose these RNases are as follows: two cold‐shock domains (CSD1 and CSD2), the RNase II catalytic domain (RNB) and a S1 domain [59] (Fig. 2). CSD1, CSD2 and S1 are considered oligosaccharide/oligonucleotide binding fold (OB‐fold) domains, which means they assist in RNA binding [59]. The RNB domain, responsible for the 3′–5′ exonucleolytic degradation of RNA molecules [60], is highly conserved. Its name arises from the *E. coli rnb* gene that encodes the family prototype RNase B (renamed as RNase II). In fact, the RNB domain is considered the main characteristic of this family, giving it the name.

While this arrangement is shared by prokaryotic RNase II and RNase R, the eukaryotic Dis3/Rrp44 and Dis3L1 evidence two additional N‐term domains, both related to the association of these proteins with the RNA exosome: the CR3 and the PilT N‐terminus (PIN) domains [61, 62, 63, 64] (Fig. 2). This last domain has a dual function, since it is required for interacting with the RNA exosome and confers endoribonucleolytic activity, enabling the enzyme to cut circular and single‐stranded RNAs (circRNAs and ssRNAs, respectively), in a Mn^2+^‐dependent manner. This second feature relies on four essential residues in the PIN domain active site (E97, D69, D177 and D146 in the human Dis3 homologue, hDIS3). However, while Dis3 can cleave RNA molecules endoribonucleolytically, Dis3L1 harbours an inactive PIN domain due to the absence of two of the four active site residues (E97 and D146 in hDIS3), making it unable to cleave RNAs internally.

In Dis3L2, the CR3 and PIN domains are missing, so this enzyme acts independently of the RNA exosome in an alternative RNA decay pathway (Fig. 2). This autonomy was confirmed in *Schizosaccharomyces pombe* [40], by the absence of its colocalization with the nuclear RNA exosome. Also, in human cell lines [41, 43], Dis3L2 did not coprecipitate with any of the subunits of the exosome (e.g. Dis3 or Rrp6).

Dis3 is mainly part of the nuclear RNA exosome, and Dis3L1 plays its catalytic role in the cytoplasmic isoform of the RNA exosome [65, 66]. In turn, Dis3L2 is found in the cytoplasm [40, 41, 42, 43] and in cytoplasmic foci anchored to processing bodies (P‐bodies) [40]. Besides their differential spatial distribution in the cell, it is also possible to distinguish these eukaryotic homologues according to their sequence conservation in the RNB active site [40]. Their amino acid signature might be DIDD (for Dis3 proteins), DVDD (for Dis3L1 enzymes) or DLDD (in the case of Dis3L2 RNases) [40]. Moreover, the aspartic acids (D) contained in these signatures are also engaged in the four aspartic acids that are critical for exoribonuclease activity, including an aspartic acid 6 nt upstream from these three. The positions of these residues correspond to D479, D485, D487 and D488 in the hDIS3 protein.

In relation to their catalytic features, the homologues differ slightly in the size of the final degradation products they generate: a minimum of 3 or 4 nt for Dis3 orthologues (budding yeast Rrp44 and hDIS3, respectively) [10, 66, 67]; 4 nt for Dis3L1 orthologues [66]; and 3 nt for Dis3L2 orthologues [40, 42].

There is not yet evidence of the ability of Dis3L1 to cleave double‐stranded RNAs (dsRNAs), but it has already been reported that Dis3 can degrade structured RNAs, as long as they bear a single‐stranded 3′ overhang with at least 4 nt (corroborated both in budding yeast and in human Dis3 orthologues) [37, 42, 66, 67]. Surprisingly, studies based on Dis3L2 orthologues from *S. pombe* (SpDis3L2) and humans (hDIS3L2) demonstrated that these RNases just require two protruding nucleotides at the 3′‐end of the structured RNAs in order to unwind them, and even manage to degrade them if they have blunt ends [40, 42].

A recent study on the landscape of functional interactions of human processive RNases, including Dis3‐like enzymes, identified new functional interactions between them. Despite the limited interpretation of data, this study provided insights into the multiplicity of processes in which these enzymes are involved and how cellular processes can be interrelated [68].

The involvement of hDIS3, and to a smaller extent hDIS3L1, in human disease is well established (as previously reviewed in [14, 15]). In this review, we give more detail on what is known regarding the association of the more recently discovered third homologue, hDIS3L2, with disease.

## The importance of hDIS3L2 in human disease

Considering the diversity of processes in which Dis3L2 is engaged, it is conceivable that it is intimately related to human health and disease. The human Dis3L2 gene (*hDIS3L2*) is annotated in chromosome 2, and it has fourteen possible splicing variants and five confirmed isoforms. The canonical isoform 1 possesses 21 exons, resulting in 885 amino acids and a molecular weight of 99.3 kDa. Interestingly, when consulting the Human Protein Atlas (HPA) platform, it is possible to ascertain that the protein ‘DIS3 mitotic control homolog‐like 2’ has detectable RNA expression in all analysed cell types, cell lines and tissues (including the brain), but protein expression levels vary among tissues [69].

In Table 1, we have presented details of the *hDIS3L2* mutations that have been linked with human diseases and how they impact on its enzymatic function. The corresponding position of these amino acid substitutions is indicated in Fig. 2. Germline mutations in *hDIS3L2* have been associated with PRLMNS, a rare congenital overgrowth disease, with high neonatal mortality [51, 70, 71, 72]. Because it has autosomal recessive inheritance, the PRLMS only manifests itself if there is a biallelic abnormality in the gene that encodes hDIS3L2. A few distinct heterozygous genomic variants have been identified [51, 70], although most cases appear to be related to homozygous deletion of exon 6 or 9 [51, 71] (Table 1). Newborns suffering from this syndrome usually have numerous fetal malformations, including altered facial features, neurological delays, organomegaly and renal abnormalities. The last feature may translate into nephroblastomatosis, a precursor of nephroblastoma, also known as Wilms' tumour (WT) [73]. This is a type of kidney cancer mainly found in children, particularly in infants with PRLMNS who managed to survive beyond the neonatal period. In a study that examined sporadic WTs, 30% of them were proved to have a partial or complete deletion of the *hDIS3L2* gene [51]. A recent study was published revealing the first insights into the mechanism behind this overgrowth phenotype in humans and flies [48].

**Table 1:** Details regarding *hDIS3L2* mutations that have been linked with the human diseases listed and how they impact on the enzymatic function.

Disease in study	Alleles	Type	Region	Nomenclature			Impact on hDIS3L2	Source	
				Transcript, c.^a^	Polymorphism (dbSNP, NCBI)	Predicted effect (Protein, p.^b^)			
PRLMNS	Homozygous	Frameshift deletion	e6	c.367‐41553_ 602+40962del	n.a.	p.Val123Glufs*136	Loss of CDS2, RNB, S1 (258 aa)	[51]	
	Homozygous	In‐frame deletion	e9	c.951‐?_1124+?del	n.a.	p.Gln318_Arg375del	Loss of RNB and miR‐562 binding region		
	Heterozygous	In‐frame deletion	e19	c.2394+5G>A (in i19^c^)	n.a.	p.Glu764_Asn798del	Partial loss of RNB, S1		
		Mutation	i	c.2289+37G>A	–	Not expressed	Not expressed		
	Heterozygous	In‐frame deletion	e9	c.951‐?_1124+?del	–	p.Gln318_Arg375del	Loss of RNB and miR‐562 binding region		
		Missense mutation	e13	c.1466G>A	rs387907116	p.Cys489Tyr^NV.PS (1)^	Single aa substitution in RNB (maintain 885 aa)		
	Homozygous	In‐frame deletion	e9	m.i.	n.a.	p.Gln318_Arg375del	Loss of RNB and miR‐562 binding region	[71]	
	Heterozygous	Mutation	i5^c^	c.367‐2A>G	–	p.Val123Leufs*154	Premature STOP codon after 32 aa, losing CSD1, CSD2, RNB, S1	[72]	
		Mutation	e12	c.1328T>A	–	p.Met443Lys^NV.PS (2)^	Preserve CSD1, CSD2, S1 and RBN with single aa substitution (885 aa)		
OFCs and CTEV	Heterozygous	Missense Mutation	e8	c.878C>A	rs187563594	p.Pro293His	Likely deleterious and damaging	[70]	
		Missense mutation	e13	c.1570G>A	rs201308521	p.Glu524Lys	Likely deleterious and damaging		
n.a.	n.a.	Missense mutation	e2	c.34C>A or c.34C>T	rs723044	p.Pro12Ser^NV‐U^	Maintain 885 aa		UniProt [168]
		Missense mutation	e13	c.1447C>G or c.1447C>T	rs186865544	p.Arg483Gly^NV‐U.WT^	Maintain 885 aa		
		Missense mutation	e14	c.1727G>A	rs200386096	p.Arg576His^NV‐U.WT^	Maintain 885 aa		
MLS	Heterozygous	Deletion	Chr 2	2q37.1q37.3 deletion of 4.5 Mb	n.a.	m.i.	Predicted breakpoint at intron 6 of hDIS3L2, eliminating a 3′‐end portion of hDIS3L2 + 45 genes		[169]
		m.i.	m.i.	m.i.	m.i.	m.i.	m.i.		
ATC	m.i.	Missense mutation	e9	c.1082G>T	–	p.Trp361Leu	Likely tolerated, damaging or disease causing		[77]

OFCs, orofacial clefts; CTEV, congenital talipes equinovarus (or clubfoot) – constitutes a rare possible manifestation of PRLMNS, although the remaining common symptoms of PRLMNS were not described in the patient of the referred study; Region: genomic region affected – might be a chromosome (Chr), or a particular exon (e) or intron (i) of hDIS3L2 protein; n.a.: not applicable; m.i.: missing information (not mentioned on the original source); aa: amino acid. ^NV.PS^natural variant of the protein in PRLMNS (manually annotated in UniProt); ^(1)^p.Cys489Tyr (C489Y) assigned as pathogenic variant (ClinVar Accession Allele A: RCV000024121.3), being a substitution in a conserved residue, including in *Schizosaccharomyces pombe*, *Drosophila melanogaster*, *Arabidopsis thaliana* and *Mus musculus*; ^(2)^p.Met443Lys (M443K) has unknown pathological significance, although possessing this mutation in at least one of the alleles enables long‐term survival (due to retention of partial exonucleolytic activity); ^NV^natural variant of the protein (manually annotated in UniProt), apart from the two NV.PS; ^U^NV with unknown clinical implication in PRLMNS; ^U.WT^NV with potential association with the pathogenicity of PRLMNS (still uncertain) was found in a patient with WT.

^a^ In case of a known mutation, the denotation ‘c.n°A>B’ means that, in the coding DNA reference sequence, the nucleotide A in position n° was replaced by nucleotide B

^b^ In case of a known single amino acid substitution, the denotation ‘p.An°B’ means that, on protein level, the reference amino acid A in position n° was modified to an amino acid B

^c^ Splice‐site mutation.

In this syndrome, it is curious to notice that the deletion in exon 6 resulted in a distinct subcellular expression pattern of hDIS3L2, with the enzyme being additionally found in the nucleus [51]. A similar modification in subcellular localization was seen in patients with intranuclear inclusion body disease [74]. This is a neurodegenerative disease in which one of the hallmarks is the occurrence of nuclear inclusions in neurons and glial cells, which frequently contain proteins related to amyotrophic lateral sclerosis. In addition, these nuclear inclusions were found to retain the exoribonucleases XRN1 and hDIS3L2, which may therefore be responsible for their formation or degradation [74].

Moreover, it was also discovered that patients with Marfan‐like syndrome (MLS) display a chromosomal translocation that results in partial deletion of the *hDIS3L2* gene, abrogating its exonucleolytic activity (reviewed in [75]) (Table 1). In turn, this *hDIS3L2* truncation is related to the overexpression of C‐type natriuretic peptide (CNP), encoded by the gene *NPPC* that regulates bone growth, subsequently leading to excessive bone growth and skeletal malformations, which are characteristic of this syndrome.

Mutations in *hDIS3L2* have also been connected to various human cancers, namely colorectal cancer [76], anaplastic thyroid cancer (ATC) [77] (Table 1), hepatocellular carcinoma [78] and testicular germ cell tumour [79]. One of the most studied pathways that might be, in some cases, related to the involvement of Dis3L2‐mediated RNA decay in the tumorigenesis process is the Lin28–let‐7 pathway. Lin28 is a pluripotency factor that negatively regulates the expression of let‐7 microRNA (miRNA), which, in turn, functions as a tumour suppressor [80]. A group of precursors of let‐7 miRNA (pre‐let‐7) must be monouridylated by the human terminal uridylyltransferases (TUTases) 4 or 7 (TUT4/7) to enable their processing by the endoribonuclease Dicer [81]. However, when Lin28 is being expressed, this factor promotes instead the oligouridylation of pre‐let‐7 by the same TUTases, TUT4/7, which, this time, has an opposite effect. The addition of a longer U‐tail prevents miRNA maturation and rather recruits Dis3L2 to rapidly degrade those precursors [41, 43, 46]. This way, the expression of let‐7 targets is allowed, favouring pluripotency, and eventually cell reprogramming and cancer development [81, 82]. Nonetheless, the precise role of Dis3L2 in the progression of these diverse types of cancer is still incompletely understood.

## The importance of bacterial RNases from the RNase II/RNB family in the physiology of human pathogens

Members of the RNase II/RNB family of enzymes also perform important functions in bacterial cells, and in some cases, they can be essential for growth and viability [4, 83, 84]. As already mentioned, bacteria encode two members of this family of enzymes: RNase II and RNase R. RNase II is the prototype of the family and responsible for 90% of the hydrolytic activity in cell extracts [85]. However, RNase R seems to play a major role in the physiology of several pathogenic bacteria and has been implicated in their virulence mechanisms (Table 2). Moreover, RNase R activity is modulated by environmental conditions, namely cold‐shock [86]. During adaptation to the cold, most protein synthesis stops. However, a few proteins, such as RNase R, have their expression levels significantly increased (reviewed in [87]). It is known that stabilization of RNA secondary structures occurs at low temperatures. Strikingly, RNase R is capable of degrading highly structured RNA molecules due to an intrinsic helicase activity [88], which contributes highly to the importance of this enzyme under cold‐shock conditions.

**Table 2:** Role of RNB/RNase II family of enzymes in the virulence of pathogenic bacteria.

Bacterium	Human disease	Role of RNase R in virulence	Source
*Aeromonas hydrophila*	Diarrhoea	Needed for survival in the cold, motility; modulates virulence in mice	[89]
*Campylobacter jejuni*	Gastroenteritis	Needed for adhesion and invasion of eukaryotic cells	[90]
*Helicobacter pylori*	Peptic ulcers	Role in environmental sensing (e.g. pH, temperature) motility, expression of apoptosis‐inducing genes	[91]
*Shigella flexneri*	Dysentery	Influences the expression of invasion and virulence factors, adhesion and capacity to spread inside eukaryotic cells	[93, 94]
*Legionella pneumophila*	Legionnaires' disease (severe pneumonia)	Needed for growth in the cold; the absence of RNase R induces competence	[96]
*Mycoplasma genitalium*	Nongonococcal urethritis; pelvic inflammatory disease	Essential enzyme; important for RNA degradation and RNA processing (tRNA maturation)	[100, 101, 102]
*Streptococcus pneumonia*	Pneumonia, meningitis, otitis media and septicaemia	Affects translation; overall decreases protein synthesis; is upregulated under cold shock; plays an important role in pathogenicity	[125, 126, 167]

In recent years, research into RNA metabolism and RNases in foodborne and gastrointestinal pathogens has increased considerably. Most foodborne pathogens can withstand refrigerated temperatures, and the role of RNase R under cold‐shock conditions led to studies targeting this RNase. In *Aeromonas hydrophila*, an enteric pathogen, RNase R, was demonstrated to be crucial for cell survival at low temperatures [89], confirming the role of this protein under cold‐shock conditions. This protein was also shown to be crucial for cell motility and to modulate *A. hydrophila* virulence in mice [89] (Table 2). *Campylobacter jejuni* is the major global cause of foodborne gastroenteritis. It encodes an RNase R homologue, which was shown to be active under a wide range of conditions, a feature that could be important for adaptation during the infection process [90]. Loss of RNase R results in a decreased capability to adhere and invade eukaryotic cells [90] (Table 2). *Helicobacter pylori* is considered to be a major risk factor for the development of gastric cancer. In this bacterium, RNase R plays an important role in environmental sensing and in the regulation of virulent phenotypes, such as motility and apoptosis of the host cells [91] (Table 2). More recently, it was described that RNase R from *H. pylori* does not have helicase activity (contrary to what was described in *E. coli*) and forms a functional complex with RhpA, a DEAD‐box RNA helicase, to better degrade structured substrates [92]. *Shigella flexneri*, which is the causative agent of dysentery in humans and monkeys, also relies on RNase R for its virulence. Namely, RNase R was shown to influence the production of virulence‐associated antigens at the post‐transcriptional level (IpaB, IpaC, IpaD and VirG). It also affected the capacity of bacteria to adhere and spread inside cells [93, 94] (Table 2). *Legionella pneumophila* is a ubiquitous bacterium in freshwater environments and in several manmade water systems that causes a potentially lethal pneumonia: Legionnaires' disease. Therefore, this bacterium needs to survive at a wide range of temperatures, including the low temperatures of air conditioners [95]. *Legionella pneumophila* codes for an RNase R homologue, which was also shown to be important for cell growth at lower temperatures [96]. In the absence of RNase R, there is an accumulation of unprocessed and structured RNA molecules under cold‐shock conditions [96], confirming the crucial role that this enzyme has in the degradation of highly structured RNA molecules at lower temperatures. Moreover, at its optimal temperature of growth, the absence of RNase R induces competence development in *L. pneumophila* (Table 2). This was the first time that the absence of RNase R was reported to result in a gain of function in an organism [96]. More recently, it was described that an *E. coli* K12 strain lacking RNase R also gained the ability to metabolize propionate under anoxic conditions [97]. In the plant pathogen *Pseudomonas syringae*, the absence of RNase R also impairs growth at low temperatures [98]. Moreover, in this bacterium, RNase R (and not PNPase, as is more typical) is part of the degradosome, a multiprotein complex involved in mRNA degradation [99]. The examples described above confirm the critical role that RNase R plays in the cells, namely under cold conditions. However, the functions of this enzyme are even broader. In *Mycoplasma genitalium*, RNase R is the only exoribonuclease present in the cell, and thus, it is an essential protein. It is able to degrade a panoply of RNA substrates; however, it is sensitive to certain structural features and ribose methylation [100, 101]. Besides its role in RNA degradation, RNase R from *M. genitalium* was also implicated in RNA processing, namely transfer RNA (tRNA) maturation [101, 102] (Table 2). The ability of RNase R from *M. genitalium* to discriminate between 2′‐*O*‐methylations can be further used as a powerful analytical tool for the research of RNA modifications in biological and clinical samples [103].

Besides its important role in pathogenesis, RNase R is also involved in other important processes in the cell. It was demonstrated that RNase R interacts with ribosomes [104, 105], namely with the 30S subunit [105] and with the S12 protein [104, 106, 107]. This association was also described in the important human pathogen *Staphylococcus aureus* [108]. RNase R is indeed stabilized when it is bound to the ribosomes, which allows control of its enzymatic activity. While bound to ribosomes, RNase R is not available to degrade functional RNA molecules [104]. Also, in coordination with the endoribonuclease YbeY, RNase R can cleave defective ribosomes *in vitro*, demonstrating the important role that this protein plays in quality control pathways [109]. Reinforcing this, RNase R, together with PNPase, was also shown to be involved in rRNA quality control [110, 111]. More recently, in a coordinated action with PNPase and RNase E, RNase R was implicated in the rifampicin‐induced rRNA degradation that occurs under different growth conditions [112].

Another example that reinforces the importance of RNase R in quality control is its role in the *trans*‐translation pathway. *Trans*‐translation is a ubiquitous bacterial mechanism that resolves ribosome stalling caused by nonstop mRNAs. In *trans*‐translation, deficient proteins and mRNA molecules are guided for degradation, and stalled ribosomes are rescued. It was discovered that RNase R is important for the maturation of transfer‐mRNA (tmRNA) in *E. coli* under cold‐shock conditions [86]. Furthermore, it was later shown that RNase R is the RNase responsible for the degradation of mRNA molecules during *trans*‐translation [113]. It was described that RNase R from *E. coli* associates with a ribonucleoprotein complex that contains an RNA molecule named tmRNA and the RNA‐binding protein SmpB, reinforcing the role of RNase R in *trans*‐translation (reviewed in [114]). The *trans*‐translation pathway is an attractive target for antibiotics since a functional ribosome is required to maintain bacterial viability [115, 116, 117, 118, 119, 120, 121]. Therefore, the interconnection of RNase R with the *trans*‐translation components is relevant and was observed in several bacteria [122, 123, 124, 125].

Besides its role in protein quality control, it was also shown that RNase R from *Streptococcus pneumoniae* is involved in translation by affecting the amount of 70S ribosomes required for proper mRNA translation, and by regulating the expression level of important translation factors implicated in ribosome recycling [126] (Table 2).

Considering the crucial role of RNase R, it is expected that the levels of this protein must be tightly controlled. Indeed, RNase R levels increase in cold‐shock conditions, as already addressed above, but the enzyme can be regulated in other ways. In the stationary phase, although there is a decrease of its mRNA levels (*rnr* message), there is an increase in the amount of RNase R protein in the cell [127]. This is due to tight regulation of RNase R stability. When cells are in the exponential phase, RNase R is acetylated and marked for degradation. In contrast, when cells reach the stationary phase of growth, the protein responsible for the acetylation is no longer expressed and RNase R is highly stabilized [124, 128]. Interestingly, this regulation is not observed in *S. aureus* [129], which may indicate that this is not a conserved mechanism. It was shown that the activity of RNase II, the prototype of this family of enzymes, is also modulated post‐translationally through acetylation of the Lys501 amino acid. Although the acetylation step causes a decrease in RNase II activity, its stability is not compromised [130]. The stability of RNase II was shown to be regulated by *gmr* (gene‐modulating RNase II), on the basis that RNase II protein turnover is slower in the *gmr* deletion strain [129]. Furthermore, RNase II levels are also regulated depending on the growth media, and this regulation is abolished in a strain lacking *gmr* [129]. This shows that the regulation of ribonucleolytic activity can depend on growth conditions and that this regulation can be mediated by alternative factors that are not RNases.

## The importance of SARS‐CoV‐2 viral 3′–5′ exoribonucleases to combat the COVID‐19 pandemic

In mammalian cells, exoribonucleases are also involved in the fight against potential pathogenic microorganisms, such as viruses. For instance, exoribonucleases involved in nonsense‐mediated decay (NMD) were shown to impact several RNA viruses, such as the model coronavirus mouse hepatitis virus (MHV). Yet, the opposite also happens when NMD is inhibited upon viral infection [131, 132]. Therefore, the interplay between viruses and host cells is critical to determine the fate of an infection.

In the context of the current COVID‐19 pandemic caused by the severe acute respiratory syndrome coronavirus 2 (SARS‐CoV‐2), particular attention was given to its 3′–5′ hydrolytic exoribonucleolytic activity. This enzymatic activity was found to be highly conserved within the *Coronaviridae* family and to play essential roles in the life cycle of coronaviruses (CoVs) [133]. This activity is provided by the nonstructural protein 14 (nsp14), which is a very peculiar and interesting enzyme, since its N‐terminal region (amino acids 1–290) confers the 3′–5′ exoribonuclease activity (ExoN) and the C‐terminal (amino acids 291–527) folds into a noncanonical methyltransferase (N7‐MTase) [133, 134, 135, 136, 137]. The fact that nsp14 is a bifunctional enzyme demonstrates the complexity of CoV proteins. Although N7‐MTase has a critical role in the CoV life cycle, this section will only highlight the role of the exoribonucleolytic activity of nsp14 ExoN in SARS‐CoV‐2.

CoVs contain by far one of the largest and most complex RNA virus genomes (approximately 30 kb). The nsp14 ExoN domain belongs to the DEDD 3′–5′ exonuclease superfamily, which includes the proofreading domains of many DNA polymerases [135, 138]. Therefore, these viruses can preserve a replication‐competent genome, because the ExoN activity of nsp14 plays a proofreading role to enhance replication fidelity and avoid lethal mutations [139]. Nsp14 proofreading activity is also known to excise nucleoside analogues (NAs), a class of drugs widely used as a treatment for several viral infections [140, 141, 142, 143, 144]. NAs are incorporated into the elongation chain to stall RNA synthesis. However, their effectiveness is much higher against viruses that do not possess proofreading activity [145]. Indeed, β‐coronavirus with inactivated ExoN activity are profoundly more sensitive to a range of NAs and therefore induces susceptibility to certain antivirals, such as remdesivir (GS‐57349) [143, 146, 147].

Despite the existence of vaccines, there is concern about the threat posed by the emergence of new SARS‐CoV‐2 variants. The generation of these variants might be explained through the occurrence of genomic recombination events [148]. Paradoxically, the nsp14 ExoN domain, which is responsible for maintaining a low mutation rate during replication, was found to be associated with this recombination potential for repair or escape [148]. Thus, nsp14 can be considered a promising target to compromise the ability of SARS‐CoV‐2 to recombine, and to overcome the vaccine escape reported for multiple CoVs [149, 150].

The pleiotropic nonstructural protein 10 (nsp10) was found to form a heterodimer with the ExoN domain of nsp14 to stabilize it and stimulate its activity, similar to what was verified in other CoVs. Therefore, nsp14 relies on nsp10 for its ExoN activity [133, 136, 137, 151, 152, 153]. Saramago *et al*. [137] identified critical amino acids from the SARS‐CoV‐2 nsp14‐nsp10 complex that can be used as targets for the design of effective drugs. Attacking these residues significantly reduces or blocks viral 3′–5′ exoribonucleolytic activity. Although SARS‐CoV‐2 nsp14 ExoN shows high sequence identity with the SARS‐CoV‐1 homolog (~ 95%), some differences were found for the functionality of nsp14 ExoN catalytic residues, which can be related to the particular pathogenesis of SARS‐CoV‐2 [137]. Strikingly, SARS‐CoV‐2 with the ExoN domain inactivated was found to be unable to replicate, while ExoN knockout mutants of the closely related SARS‐CoV are viable, even with an increased mutation frequency [136]. This shows the essential nature of the ExoN domain in this novel coronavirus.

The ExoN activity of SARS‐CoV‐2 nsp14 is assisted by a two‐metal ion mechanism; different divalent ions modulate its activity, with specific selectivity towards Mg^2+^ and Mn^2+^, although maximal activation is observed with Mg^2+^. The ions Co^2+^ and Zn^2+^ promote residual activity, and catalysis is not supported by Ca^2+^, Ni^2+^ or Cu^2+^ [137, 154, 155]. This demonstrates that the surrounding environment may have an influence on the viral exoribonucleolytic activity. Alteration in ion homeostasis in favour of infection has been already demonstrated in several viral systems, including SARS‐CoV [156]. Recently, serum levels of metal elements, such as Zn^2+^, were considered helpful in the evaluation of the dynamic of COVID‐19 infection [157].

In fact, nsp14 protein is a multitasking enzyme, not only involved in viral life cycle but also capable of interfering with host cells, namely with hindering the host innate immune system. Its ExoN activity cleaves double‐stranded RNA intermediates that are generated during viral replication, which would otherwise activate the type I interferon (IFN‐I) response [158, 159, 160]. The SARS‐CoV‐2 nsp14 ExoN domain was also shown to shut down cellular protein synthesis, abolishing the host IFN‐I response [161]. Indeed, nsp14 was identified as one of the most potent interferon antagonists from SARS‐CoV‐2, thus contributing to induce a delayed IFN‐I response [162]. In the absence of effective IFN expression, SARS‐CoV‐2 is able to replicate to higher titres, resulting in an exaggerated inflammatory response that leads to morbidity and mortality in patients [163].

SARS‐CoV‐2 has an exceptionally high level of transmissibility and adaption to new conditions. This has been translating into an exorbitant number of infections and deaths worldwide. The existence of nsp14 3′–5′ exoribonuclease activity largely contributes to this, since it is a key component of recombination and high‐fidelity replication, and contributes to immune system evasion, virulence and resistance to antivirals.

## Conclusion

The major features of the exoribonucleases from the RNase II/RNB family impact human disease both directly and indirectly. The eukaryotic Dis3 and Dis3L1 are known to interact with the exosome complex, but in different cellular locations. They can act on a wide range of substrates in eukaryotic cells, and their direct involvement in cancer development and progression is also well documented (mainly for hDIS3) [14, 15, 16, 164]. Beyond these, other exosome components have been also linked to human diseases (reviewed in [165]). Dis3L2, the third homologue of Dis3‐like enzymes, was later identified as a major player in the 3′–5′ exonucleolytic decay of transcripts [40]. Unlike the other two homologues, it does not associate with the exosome and leads to an alternative and relevant pathway of eukaryotic RNA decay [40]. Markedly, the discovery of its preference for uridylated RNA substrates unveiled the importance of terminal uridylation in eukaryotic RNA decay [166]. There is a strong link between hDIS3L2 and human disease [41, 51]. In this review, we described the recent discoveries of the mechanism of action and specific RNA targets of Dis3L2, and the consequences of the impairment of its enzymatic activity on the physiology of human cells.

The role of the bacterial counterparts of RNB family in disease was also reviewed. The interaction of pathogenic bacteria with host immune cells triggers a series of events that include rapid genetic reprogramming. In this context, RNases act as key modulators of RNA levels for this rapid adaptation and have been shown to be virulence‐associated factors. Prokaryotic RNases have been shown to modulate the virulence and performance of many human pathogens. The gene encoding RNase R was the first to be linked with bacterial virulence [93, 94]. However, the role of this enzyme in pathogenicity is inconsistent among different Gram‐negative bacteria. RNase R seems to significantly affect the virulence pattern of certain pathogens, but this is not the case for all of them. This may be related to the fact that each pathogen may use different molecular strategies to subvert the complex pathways that regulate the host immune response.

In the context of the current COVID‐19 pandemic caused by the RNA virus SARS‐CoV‐2, we considered it was also important to discuss the importance of the role of a viral hydrolytic 3′–5′ exoribonuclease in viral infection. The viral protein nsp14 harbours 3′–5′ exoribonucleolytic activity, is responsible for high‐fidelity RNA synthesis, and also confers resistance to NAs, leads to immune evasion and contributes to virulence [137].

Since bacterial and viral exoribonucleases seem to be necessary for the virulence of many pathogens, their study will be a stepping stone for the development of novel antibacterial and antiviral therapeutics that target them and impair their activity.

All the evidence presented here, as two perspectives of the same story, reinforces the message that the understanding of RNase II/RNB family of RNases in RNA metabolism, both in microbes and in higher organisms, is required to identify and combat many mechanisms that lead to human disease.

## Conflict of interest

The authors declare no conflict of interest.

## Author contributions

SMC, MS, RGM and SCV wrote the original draft. SMC and MS designed the figures. SCV and CMA conceptualized and coordinated the work and edited the final version of the manuscript. All authors contributed to the discussion of the work and final revision, and approved the final manuscript.
